# A promiscuous ancestral enzyme´s structure unveils protein variable regions of the highly diverse metallo-β-lactamase family

**DOI:** 10.1038/s42003-021-01671-8

**Published:** 2021-01-29

**Authors:** Pablo Perez-Garcia, Stefanie Kobus, Christoph G. W. Gertzen, Astrid Hoeppner, Nicholas Holzscheck, Christoph Heinrich Strunk, Harald Huber, Karl-Erich Jaeger, Holger Gohlke, Filip Kovacic, Sander H. J. Smits, Wolfgang R. Streit, Jennifer Chow

**Affiliations:** 1 Department of Microbiology and Biotechnology, University of Hamburg, Ohnhorststrasse 18, 22609 Hamburg, Germany; 2 Center for Structural Studies (CSS), Heinrich Heine University Düsseldorf, Universitätsstrasse 1, 40225 Düsseldorf, Germany; 3 Institute of Molecular Enzyme Technology (IMET), Heinrich Heine University Düsseldorf, 52426 Jülich, Germany; 4 Institute for Microbiology and Archaeal Center, Regensburg University, 93035 Regensburg, Germany; 5 Institute of Bio- and Geosciences IBG-1: Biotechnology, Forschungszentrum Jülich GmbH, 52426 Jülich, Germany; 6 John von Neumann Institute for Computing (NIC), Jülich Supercomputing Centre (JSC) & Institute of Biological Information Processing (IBI-7: Structural Biochemistry), Forschungszentrum Jülich GmbH, 52425 Jülich, Germany; 7 Institute for Pharmaceutical and Medicinal Chemistry, Heinrich Heine University Düsseldorf, 40225 Düsseldorf, Germany; 8 Institute of Biochemistry, Heinrich Heine University Düsseldorf, 40225 Düsseldorf, Germany

**Keywords:** X-ray crystallography, Archaeal biology, Biocatalysis, Molecular evolution, Hydrolases

## Abstract

The metallo-β-lactamase fold is an ancient protein structure present in numerous enzyme families responsible for diverse biological processes. The crystal structure of the hyperthermostable crenarchaeal enzyme Igni18 from *Ignicoccus hospitalis* was solved at 2.3 Å and could resemble a possible first archetype of a multifunctional metallo-β-lactamase. Ancestral enzymes at the evolutionary origin are believed to be promiscuous all-rounders. Consistently, Igni18´s activity can be cofactor-dependently directed from β-lactamase to lactonase, lipase, phosphodiesterase, phosphotriesterase or phospholipase. Its core-domain is highly conserved within metallo-β-lactamases from Bacteria, Archaea and Eukarya and gives insights into evolution and function of enzymes from this superfamily. Structural alignments with diverse metallo-β-lactamase-fold-containing enzymes allowed the identification of Protein Variable Regions accounting for modulation of activity, specificity and oligomerization patterns. Docking of different substrates within the active sites revealed the basis for the crucial cofactor dependency of this enzyme superfamily.

## Abstract (web summary)

The structure of Igni18, ancient enzyme from the Crenarchaean species Ignicoccus hospitalis, is solved and characterized by Pérez-García, Chow and colleagues. This structure provides insight as to the evolution of metallo-beta-lactamases and their functions.

## Introduction

Metallo-β-lactamases (MβLs) form a very extensive protein family displaying a vast number of specialized enzymes such as nucleic acid hydrolases, lactonases, hydroxylases or phospholipases. MβLs are ubiquitously distributed within Eukarya, Bacteria and Archaea, which indicates an ancient origin of this protein superfamily^1–4^. For this reason, MβLs are often studied as examples of protein evolution and ancestral sequence reconstructions^5^. The MβL-fold can be considered evolutionarily successful because it provides a large active site volume suitable for binding multiple substrates of different complexity. The cavity volume is an important factor determining enzyme promiscuity^6^. Changes in the gene sequence that occur during evolution can lead e.g. to modifications of the active site´s surrounding loops. Small changes can already have a crucial impact on the enzymes´ substrate specificity and on other traits like metal preference and catalytic efficiency^7^. Multi-drug resistant pathogens take advantage of this fast-evolving group of enzymes^2^. The enzymes UlaG (l-ascorbate-6-phosphate lactonase^8^), different RNases (RNaseZ, RNaseJ, CPSF 1 and 2^9,10^), and *N*-acylphosphatidylethanolamine phospholipase D (NAPE-PLD^11^) contain the MβL-fold. In Gram-negative bacteria, MβLs or Ambler class B β-lactamases contain one or two Zn^2+^ ions at their active site which activate(s) a nucleophilic water molecule opening the ring structure^12^. They are of great concern because they can inactivate all β-lactam antibiotics apart from monobactams.

While these enzymes apparently exert a rather specific function, Igni18, a promiscuous and primordial representative of the MβL family, could represent an example of a typical ancestral enzyme, because its structure and active site resemble the core of many modern specialized enzymes. The detailed structural analysis of Igni18, leading to the definition of Protein Variable Regions (PVRs), illustrates the evolutionary steps involved in acquiring additional activities and where these specialization loops and domains get inserted in the classic MβL fold. Although natural protein evolution is generally assumed to start with a stable protein backbone and promiscuous activity, which evolves to lower stability and increasing substrate specificity^13^, a correlation between promiscuity and increased flexibility is debated^14^. Enzymes from an early point of evolution can thus serve as starting points for protein engineering approaches.

Igni18 is an enzyme of the hyperthermophilic Crenarchaeon *Ignicoccus hospitalis*, one of the most fascinating and enigmatic microorganisms known so far. *I. hospitalis* was isolated from a shallow marine hydrothermal system near Iceland and grows optimally at 90 °C^15^. The strictly anaerobic and chemolithoautotrophic organism grows on sulfur-reduction with molecular hydrogen and uses CO_2_ as sole carbon source with a novel dicarboxylate/4-hydroxybutyrate assimilation pathway^16^. *I. hospitalis* has a potential parasite, *Nanoarchaeum equitans*, which is attached to the outer membrane. While *N. equitans* can survive with a minimal genome of 490 Kbp by relying on the sustenance of its host^17^, *I. hospitalis* itself has an extremely reduced genome of 1.3 Mbp and a total number of only 1496 genes [Integrated Microbial Genomes database (IMG, https://img.jgi.doe.gov/)]^18^. Of those, ~97% turned out to be expressed in a transcriptomic study within an actively growing culture under laboratory conditions^19^. This indicates an efficient organization of the genome and of the transcription and translation machinery as almost all genes are constitutively expressed. Nonetheless, the question arises how *I. hospitalis* is able to maintain growth, propagation and metabolism not only for itself, but also for *N. equitans* with such a limited enzymatic resource.

For only 61% of the protein-coding genes of *I. hospitalis* the function was predicted, and a relatively large proportion of those is annotated as metalloenzymes (see IMG taxon ID 640753029). Metal-dependent enzymes require ions such as Mg^2+^, Ca^2+^, Mn^2+^, Fe^2+^, Co^2+^, Ni^2+^, Cu^2+^, or Zn^2+^ as cofactors for their functionality. *I. hospitalis* possesses transporters and transporter complexes for metals such as Ni^2+^ and Fe^2+^ (important for NiFe-hydrogenases), MoO_4_^-2^, Mg^2+^, Co^2+^, and probably ABC transporters for Zn^2+^ and Mn^2+^ that span across both the outer and inner membrane and the intermembrane compartment^19,20^. Until now, none of the metalloenzymes has been investigated on a structural, biochemical, or catalytic level in detail. The characterization of Igni18 conducted in this study unveiled high promiscuity and multi-functionality depending on the availability of different metal ions. This underlines the versatility of MβLs, particularly when the enzymatic equipment of the organism is limited. Further, Igni18´s structure can be regarded as the core of numerous modern MβLs from prokaryotes and eukaryotes. The detailed structural analysis exemplifies the evolutionary steps involved in acquiring additional activities.

## Results

### Recombinant Igni18 production

Igni18 was produced in the methylotrophic yeast *P. pastoris* using the vector pPICZ-A, because expression in *E. coli* failed. Briefly, for disruption of the yeast cells, the cells were thawed on ice and suspended in 5 ml lysis buffer per gram [10 mg ml^−1^ myristyl sulfobetaine (SB3-14), 1 mM phenylmethylsulfonyl fluoride (PMSF), 0.05 M NaH_2_PO_4_, and 0.3 M NaCl pH 8.0] and incubated at 70 °C for 1 h in the presence of the zwitterionic detergent SB3-14^21^. In all, 154 g of cell pellet (WW) harvested from 10 L fermentation yielded 120 mg of highly pure protein (approx. 99% purity) after His-tag purification.

Analysis under semi-native PAGE conditions revealed a single band with an apparent molecular weight of ~130 kDa (data available upon request). Under denaturing SDS-PAGE conditions, Igni18 appeared as a three-band pattern with apparent sizes of 30, 60, and 130 kDa; western-blotting confirmed the presence of His_6_-tagged Igni18 for these bands (see Kobus et al.^21^; can be provided upon request).

### Crystallization and structure determination

**Fig. 1:**
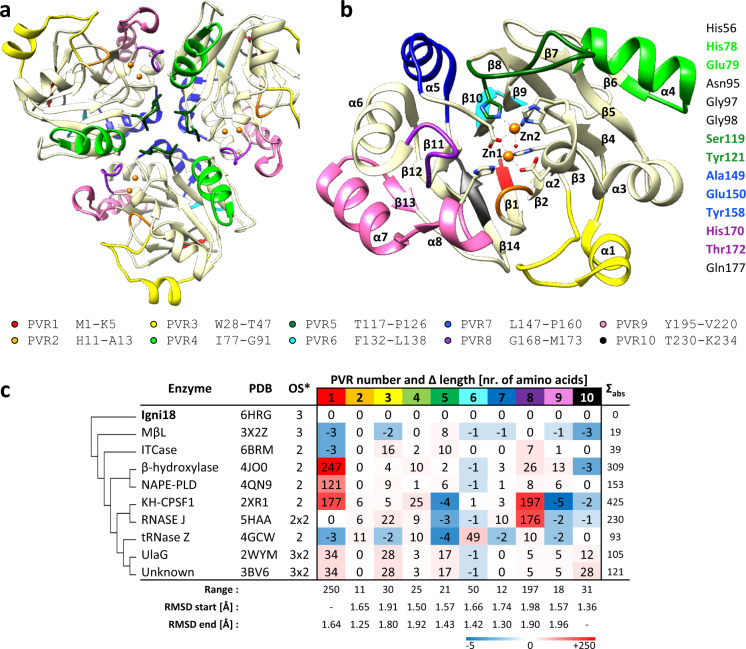
The crystal structure of Igni18 reveals the origin of diverse MβLs. The protein crystalized as a homotrimer (**a**). A monomer (**b**) comprises 8 α-helixes and 14 β-strands and contains two Zn^2+^ ions (orange). Ten Protein Variable Regions (PVRs) describe structural evolution and specialization within the MβL family and are depicted in **a** and **b**. The amino acids comprising each PVR are indicated underneath. In total, 9 out of 14 amino acids needed for stabilization of the trimer are found within PVRs 4, 5, 7, and 8 (light-green, dark-green, blue, and purple; right), leading to an evolutionary fast loss of this quaternary structure. Differences in the number of amino acids of the described PVRs among 10 MβL-fold-containing structures (**c**); Molecular relation (left) and absolute variation in the number of amino acids (Σ_abs_, right) compared to Igni18 as well as statistic data on PVR descriptors (total length range > 10 amino acids, start/end position RMSDs for all aligned structures <2 Å; bottom) are also given. Asterisk represents Oligomerization state of the crystallized enzyme (3: trimer; 2: dimer; 2 × 2: double dimer; 3 × 2: triple dimer).

Crystallization was achieved using the sitting-drop vapor-diffusion method at 20 °C with 20 mg ml^−1^ of protein in 0.1 M potassium phosphate (pH 7) and a reservoir solution consisting of 0.3 M magnesium nitrate hexahydrate, 0.1 M Tris pH 8, 22% (w/v), and PEG 8000 after several months^21^. Igni18 crystallized in space group R32 and crystals diffracted to 2.3 Å resolution. Data were collected at beamline ID30A-3 (ESRF, Grenoble, France) and processed as already described^21^. We solved the structure using a single SAD dataset. The protein model was then further built and refined to *R* values of 18.9% (*R*_work_) and 25.7% (*R*_free_) with 93.5% of the residues being in the favored region of the Ramachandran plot and 5.62% in the allowed area (see Table 1 for data collection and refinement statistics). The Igni18 coordinates were deposited in the PDB under the accession code 6HRG. Although the asymmetric unit^3^ contains only one monomer, the enzymatically active arrangement of Igni18 is a trimer, like it is also found in the crystal lattice via its symmetry-related molecules (Fig. 1a). The overall fold of each monomer is composed of two mixed β-sheets (β14, β1, β2, β3, β4, β5, β6, and β7, β8, β9, β10, β11, β12, β13), slightly twisted and arranged in a parallel fashion in the center of the protein. Those are flanked by four helices on each side of the sheets (α1, α2, α3, α4 and α5, α6, α7, α8; Fig. 1b), thereby building the four-layered αββα core typical of the MβL superfamily^22,23^. All interfaces between the three monomers have areas of ~850 Å^2^ with eleven intermolecular hydrogen bonds. Close beneath the protein surface and in direct vicinity to the loops connecting β4-α2, β10-α5, β11-α6, two Zn-ions are bound by His54, His56, His59, His118, His194, Asp58, and Asp144 (Fig. 1b and Supplementary Fig. S1).

**Table 1:** Data collection and refinement statistics (molecular replacement).

	Igni18
Data collection	
Space group	R 3 2:H
Wavelength	0.9677
Resolution range (Å)	31.32-2.3 (2.382-2.3)
* a*, *b*, *c* (Å)	67.42, 67.42, 253.77
α, β, γ (°)	90.0, 90.0, 120.0
Total reflections	70,412 (7119)
Unique reflections	10,293 (1008)
Multiplicity	6.8 (7.1)
Completeness (%)	99.78 (100.00)
Mean *I*/*σ(I)*	12.60 (3.37)
Refinement	
Wilson *B*-factor (Å^2^)	32.46
R-merge	0.1004 (0.5445)
R-meas	0.1084 (0.5872)
R-pim	0.03987 (0.2152)
CC1/2	0.998 (0.885)
CC^*^	1 (0.969)
Reflections used in refinement	10,282 (1008)
Reflections used for R-free	500 (54)
R-work	0.1730 (0.1866)
R-free	0.2108 (0.2179)
CC(work)	0.941 (0.935)
CC(free)	0.950 (0.906)
Number of non-hydrogen atoms	1908
Macromolecules	1803
Ligands	8
Solvent	97
Protein residues	233
RMS(bonds)	0.008
RMS(angles)	1.26
Ramachandran favored (%)	93.51
Ramachandran allowed (%)	5.62
Ramachandran outliers (%)	0.87
Rotamer outliers (%)	0.52
Clashscore	3.6
Average B-factor	33.9
Macromolecules	33.68
Ligands	38.59
Solvent	37.61

Values in parentheses are for highest-resolution shell.

The inductively coupled plasma-mass spectrometry (ICP-MS) analysis conducted to verify the incorporated ion revealed that Zn is preferably bound to the recombinantly produced and purified, non-stripped protein Igni18 with 87.5% mass fraction (data available upon request). Ni, Fe, and Mn accounted for 6.8, 3.9, and 1.5% (w/w) respectively. Cu (0.4% w/w) and Co were barely present or not at all. Other metals (Ti, Se, Nb, Mo, Ag, Cd, Hg, and Pb) represented only 0.45% (w/w) of the total detected mass.

### Sequence and structural similarities between Igni18 and other metallo-β-lactamases

The results obtained by amino acid sequence alignments differed greatly from the results of structural comparisons. A BLASTP search against the NCBI non-redundant database revealed homologs of Igni18 belonging to different lineages. Protein sequence comparison of Igni18 with nine of its homologs revealed high amino acid conservation within different archaeal species. The five histidines and two aspartic acids needed for metal coordination are present in all of them. Moreover, several motifs with sequence identities of more than 80% were found within the alignment (Supplementary Fig. S2).

**Fig. 2:**
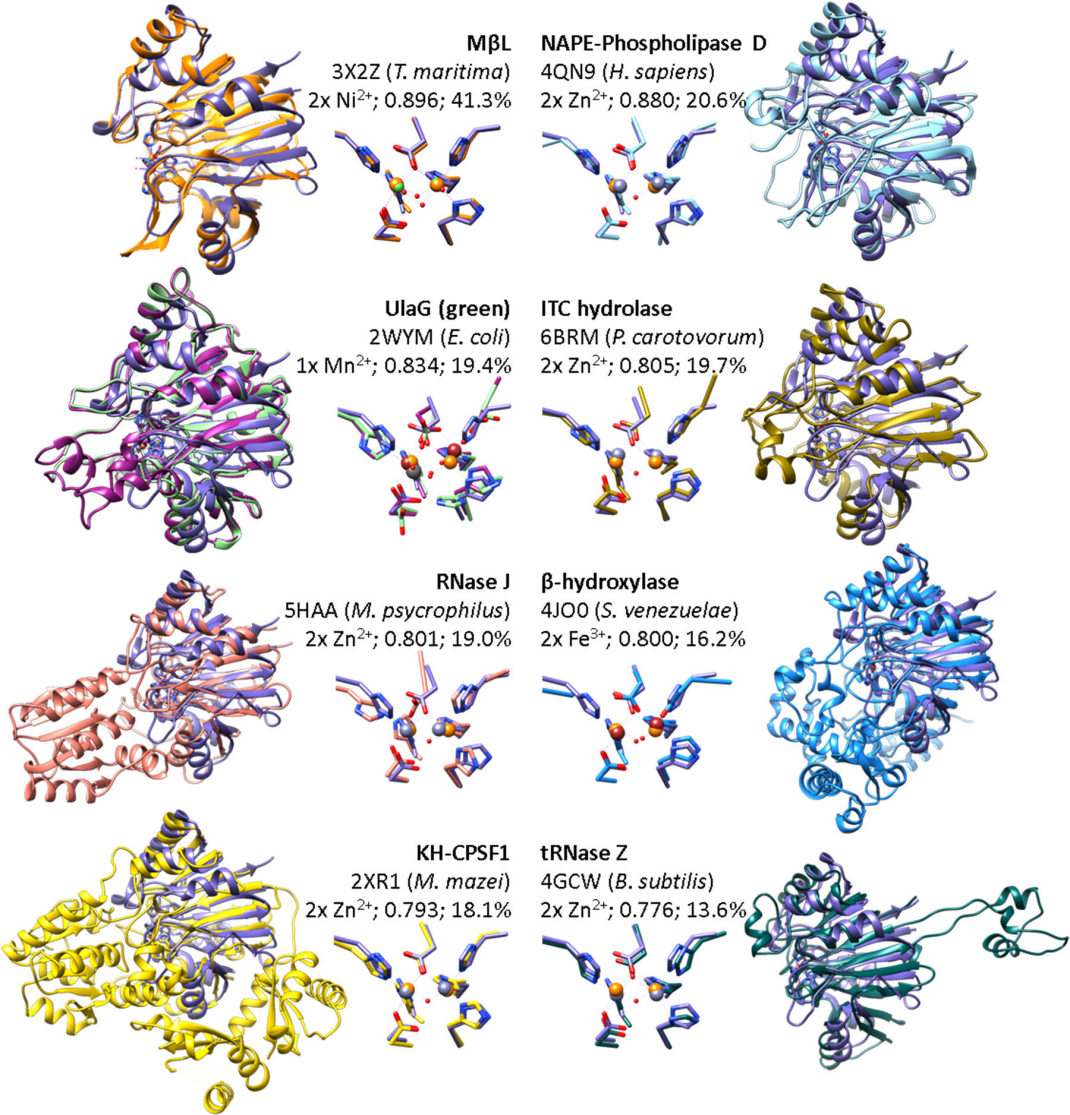
Structural conservation of the Igni18-like core-domain within MβLs with specialized activities. The homolog structures 3X2Z from *Thermotoga maritima*, 4QN9 from *Homo sapiens*, 3BV6 from *Vibrio cholerae*, 2WYM from *Escherichia coli*, 6BRM from *Pectobacterium carotovorum*, 5HAA from *Methanolobus psycrophilus*, 4JO0 from *Streptomyces venezuelae*, 2XR1 from *Methanosarcina mazei*, and 4GCW from *Bacillus subtilis* are shown. Igni18 is depicted in purple and its two Zn^2+^ ions in orange. Enzymatic function, PDB code, source organism, metal ions bound, structural identity score (TM-score), and sequence identity [%] are indicated. The uncharacterized 3BV6 protein (2x Fe^3+^; TM-score 0.8418; seq. id. 21.1 %) is shown in violet together with UlaG (in green) due to their high similarity and because of the completeness of its structure as opposed to UlaG’s. The catalytic pockets and amino acids necessary for metal coordination are shown in a close-up. Structure overlays are sorted by overall structure similarity (TM-score, Supplementary Data S1).

In contrast, structure-based searches against the PDB revealed hits belonging to very different protein families (Supplementary Data S1). In order to describe and quantify the diversity and evolution within the MβL family, a structural alignment was performed with a subset of functionally diverse proteins with more than 220 aligned amino-acids or an RMSD value below 3. This included the structures of MβL from *T. maritima* (3X2Z^24^), human NAPE-PLD (4QN9^25^), UlaG from *E. coli* (2WYM^8^), and a putative MβL from *V. cholerae* (3BV6), isothiocyanate hydrolase (ITCase) from *Pectobacterium carotovorum* (6BRM^26^), RNase J from *Methanolobus psychrophilus* (5HAA^27^), β-hydroxylase CmlA from *Streptomyces venezuelae* (4JO0^28^), CPSF1 from *Methanosarcina mazei* (2XR1^29^), and RNase Z from *Bacillus subtilis* (4GCW^30^; see Fig. 2).

The active pockets and amino acids responsible for metal coordination appear to be highly conserved (Fig. 2). As a consensus, two Zn^2+^ ions are bound by five histidines and two aspartic acids. Nevertheless, some of the analyzed proteins bind two Ni^2+^ (3X2Z), two Fe^2+^ (3BV6 and 4JO0), or one Mn^2+^ (2WYM). No differences in amino acid composition were found in the MβL from *T. maritima* (3X2Z) and the Zn-binding enzymes. The second coordination sphere of 3X2Z (His8 and Glu73′) could contribute to the specificity of Ni^2+^-binding^24^. Igni18 binds Zn^2+^ preferably, although it has these amino acids in the homologous 11 and 79′ positions (Supplementary Fig. S2). Fe^2+^-binding proteins do not contain the fourth histidine (His118 from Igni18, Fig. 1b). Instead, they possess Asp184 (3BV6) and Glu377 (4JO0). Additionally, the β-hydroxylase 4JO0 has an Ala482 instead of Igni18’s His194. UlaG (2WYM), a protein very similar to *V. cholerae*´s 3BV6, also contains an Asp184, but only binds one Mn^2+^-ion.

We defined Protein Variable Regions (PVRs) as protein sections or domains between two structurally aligned positions with RMSD smaller than 2 Å (or one position in case of the N- or C-terminal domains) and a range of length variation within all structures bigger than 10 amino acids. A total of ten MβL-PVRs were described (Supplementary Table S1), including N- and C-terminal ends (PVR1 and PVR10), three loops around the catalytic pocket (PVRs 2, 5, and 8), one between β9 and β10 (PVR6) and α1 (PVR3), α4 (PVR4), α5 (PVR7), and α7-α8 (PVR9; Fig. 1a, b). A histidine needed for metal coordination (His118) is found within PVR5 and is mutated in the Fe-binding enzyme structures 3BV6 and 4JO0 (Figs. 1 and 2). All β-strands and α2, α3, and α6 appear to be highly conserved.

PVR lengths in number of amino acids were calculated for every structure of the subset and compared to those of Igni18 (Supplementary Table S1 and Fig. 1c). All PVRs exhibited variability among the different enzyme classes, though the least diversity was found for PVRs 2, 6, 7 and 10. The MβL from *T. maritima* revealed high similarity in PVR length for all its domains but PVR5, forming two extra β-sheets. The main additions of the ITCase are found in PVRs 3, 5 (2 α-helixes) and 8, covering the access to the catalytic pocket, particularly by the lid-like structure formed from PVR3. The β-hydroxylase adds a large domain in front of the active site (PVR1 with 247 amino acids). It comprises at least seven β-sheets and thirteen α-helixes. Other remarkable modifications in the PVRs limit the access to the active site even more by adding one big loop (PVR4), two α-helixes (PVR8) and one α-helix (PVR9). The structure of the NAPE-PLD adds 121 amino acids at the N-terminus (PVR1) and contains at least two α-helixes. Again, a lid-like domain comes out of PVR3, but smaller than at the β-hydroxylase. Minor additions in PVRs 5, 8, and 9 also contribute to a slight delimitation of the catalytic site. The archaeal CPSF1 enzyme has a 177 amino acid long N-terminal PVR1. It involves two K-homology-domains (KH-domains^29^). These are involved in RNA- or ssDNA-binding and are mainly found in proteins associated with transcriptional and translational regulation. Remarkable is also the domain that comes out of PVR8 (197 amino acids) and covers the active center completely. This β-CASP domain consists of six β-sheets and nine α-helixes and is found in pre-mRNA 3’-end-processing endonucleases^9^. PVR4 adds one α-helix and extends α4. PVRs 5 and 9 are reduced, possibly facilitating the accommodation of nucleic acids. RNase J is similar to the CPSF1 enzyme but lacks KH-domains and the β-CASP domain accounts only for seven β-sheets and five α-helixes. PVRs 5 and 9 are reduced as well, while PVRs 3 and 4 are considerably larger than Igni18’s. RNase Z presents the largest variation on PVR6, a pre-tRNA binding domain (exosite) composed of three α-helixes^23^. PVR2, which is only enlarged within the RNAses, is even larger than the ones from CPSF1 and RNase J. PVR4 and PVR8 are ten amino acids larger than the ones of Igni18. UlaG presents elongations at almost every PVR, but most remarkably on PVRs 1, 3, 5, and 10 (Figs. 1c and 2).

### Enzyme activity assays for substrate promiscuity and temperature/pH optimum of Igni18

The very basic manifestation of the PVRs from Igni18 suggests a very broad spectrum of activities in comparison to its specialized MβL superfamily members. For experimental verification, an in-depth characterization on different substrates was conducted with different metal cofactors to understand the influence of these divalent ions. The compounds selected include various β-lactams, lactones and model substrates representing chemical bonds catalyzed by MβLs. The native trimer form of Igni18 was used for the assays.

#### Hydrolysis of antibiotics

**Fig. 3:**
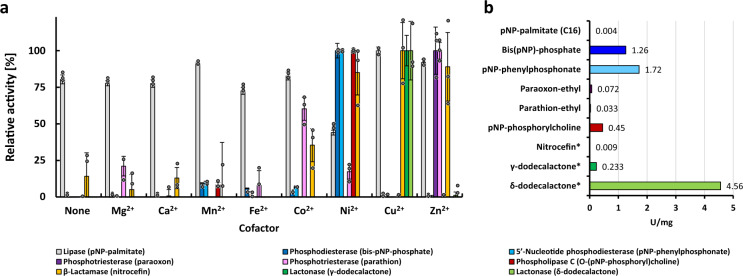
Different divalent metal ions drive Igni18’s enzymatic promiscuity. **a** Metal-dependency of Igni18 was measured with nine substrates representing activities catalyzed by MβLs. All activities but lipase showed a strict metal dependency. Error bars represent the relative standard deviation (*n* = 3 independent experiments). **b** Absolute activities measured in U mg^−1^ under optimal conditions but the ones marked with an asterisk, which were measured at 40 °C (see Methods section).

Assays for hydrolysis of heat labile β-lactams were performed at 40 °C. β-lactamase activity was mainly observed in the presence of Cu^2+^, Zn^2+^, and Ni^2+^ (Fig. 3). In the disc diffusion assay, disks containing antibiotics were incubated with purified Igni18 together with metal ions prior to performing the assay. Stability of the β-lactams was monitored with buffer controls. The disks were placed on agar coated with a fresh solution of susceptible *E. coli* DH5α wild-type cells. Enzymatic degradation of a β-lactam antibiotic resulted in the reduction of the inhibition zone (quantified in mm) around this disc after one night of incubation at 37 °C. In the experiments described below, Ni^2+^ and Zn^2+^ ions were responsible for activity on most of the different substrates tested, so they were also used in this β-lactam assay. Igni18 containing either Ni^2+^ or Zn^2+^ as cofactor showed β-lactamase activity against most of the antibiotics tested (Supplementary Fig. S3). These data confirm both the broad-spectrum activity profile of Igni18 and the flexibility of the metal-binding site in this superfamily^31^.

#### Activity profiling with substrates of non-lipolytic and lipolytic enzymes

Esterase activities of Igni18 in dependency on metal ions were assayed with various esters containing the 4-nitrophenyl [or *para*-nitrophenyl (*p*NP)] chromophore. Carboxyl-esterase and lipase activities (EC 3.1.1.1) were tested using *p*NP esters with acyl chains containing 2–18 carbon atoms, phosphatase activity (EC 3.1.3) with *p*NP-phosphate (*p*NPP), phosphodiesterase activity (PDE, EC 3.1.4) with bis-*p*NP-phosphate (bis-*p*NPP) and *p*NP-phenylphosphonate (*p*NPPP), phosphotriesterase activity (PTE, EC 3.1.8.1) with paraoxon and parathion, and phospholipase C activity (PLC, EC 3.1.4.3) with *p*NP-phosphorylcholine (*p*NPPC). Lactonase activity was measured against γ-dodecalactone and δ-dodecalactone. The pH and temperature optima of Igni18 were determined using representative substrates for each enzyme family (Supplementary Fig. S4). Lipase activity was measurable between pH 5 and 8, while the other activities were only detectable in narrower ranges. Lipase and phosphodiesterase (PDE) activities were measured at a broad temperature range between 40 °C and 95 °C, but phosphotriesterase (PTE) and phospholipase C (PLC) activities could only be measured when temperatures exceeded at least 60 °C.

Although hydrolysis of carboxyl esters was not strictly depending upon the presence of metal ions, we observed that the metal bound to Igni18 strongly influenced its PDE, PTE and PLC activities (Fig. 3a and Supplementary Data S2). Esterase activity could be measured with all substrates assayed, with long chain fatty acid esters (C12–C16) being preferred (Supplementary Fig. S4). The highest activity was measured with the lipase substrate *p*NP-palmitate. Presence of Mn^2+^, Cu^2+^, or Zn^2+^ raised esterase activity by 10–20% and Ni^2+^ lowered it by almost 50% (Fig. 3a). The enzyme showed esterase activity in the pH range of 4–8 (optimum between pH 5–7). More than 50% activity was observed over the whole temperature range tested, with a maximum at 90 °C (Supplementary Fig. S4). Phosphatase activity was not observed with *p*NPP as the substrate, whereas PDE activity was observed with both bis-*p*NPP and *p*NPPP and was strictly dependent on the presence of Ni^2+^. Presence of Mn^2+^, Fe^2+^, or Co^2+^ resulted in activities lower than 10% of the activity observed with Ni^2+^. With bis-*p*NPP and *p*NPPP, the optimum pH was found to be pH 8. Hydrolysis of bis-*p*NPP was detectable at temperatures above 50 °C, with the highest activity determined at 70 °C. The hydrolysis of the phosphotriester paraoxon was strictly Zn^2+^-dependent. Hydrolysis of the phosphotriester parathion, although occurring primarily in the presence of Zn^2+^, was also observed in the presence of Co^2+^ (60% activity) and Mg^2+^, Fe^2+^, and Ni^2+^ at rates below 25% of the maximum activity. The highest activity was found at pH 8–9 and temperatures between 90 and 95 °C. PLC activity was found to depend on Ni^2+^ as a cofactor (Mn^2+^ yielded <10%) in the temperature range from 70 to 95 °C (optimum at 90 °C) and pH 8. Finally, lactonase activity was observed when supplemented with Cu^2+^ (Fig. 3a).

The enzymatic units were measured at 90 °C under optimal pH and metal conditions with exception of β-lactams and lactones at 40 °C. The highest activities were registered with the δ-dodecalactone (4.56 U mg^−1^) and the PDE substrates bis-*p*NPP (1.26 U mg^-1^) and *p*NPPP (1.72 U mg^−1^). Igni18 showed 0.45 U mg^−1^PLC activity, 0.233 against γ-dodecalactone and only 0.07, 0.03, 0.01, and 0.004 U mg^−1^ were measured with phosphotriesterase, β-lactamase and lipase substrates, respectively (Fig. 3b and Supplementary Table S2). Michaelis–Menten kinetic parameters determined with bis-*p*NPP at 90 °C and pH 8 were *v*_max_ = 11.73E-11 mol  s^−1^; *K*_M_ = 0.0025 mM and *k*_cat_ = 6.72E-5 s^−1^.

The competition of Zn^2+^ and Ni^2+^ for binding within the catalytic pocket was studied by supplementing metal-free Igni18 with different ratios of both metals and assaying activity against bis-*p*NPP. No activity could be detected at Zn:Ni ratios 1:1 or 1:10. At 1:100 ratio, only 6% of Igni18 was active (loaded with Ni^2+^ ions) and 1:1000 yielded 53% activity. Ratios of 1:10^4^ and 1:10^5^ revealed more than 80% activity, but full activity compared to the control could only be recovered if Ni^2+^ was present in 10^6^-fold molar excess to Zn^2+^ (data available upon request).

### Binding mode prediction

As Igni18 catalyzes multiple reactions and recognizes a broad spectrum of ligands, the binding mode of these ligands is of particular interest. For a binding mode prediction, imipenem, mezlocillin, paraoxon-ethyl, and *p*NP-palmitate were chosen as they are active ligands in combination with Zn^2+^ ions for docking into the X-ray crystal structure of Igni18 using two different approaches. We only considered Zn^2+^ in the docking, as other cofactors are underrepresented in knowledge-based scoring functions. The primary docking engine used was Autodock3^32^ with DrugScore2018^33^ as a scoring function. Similarly, these ligands were docked using GlideXP^34^ while enforcing interactions to the zinc ions. However, both methods did not result in binding poses in which cleavable bonds of the ligands approached the zinc ions closer than 7 Å. These results, alongside the fact that soaking the crystals with ligands remained unsuccessful, suggest that the loops surrounding the active center were not crystallized in a conformation receptive for ligand binding.

In order to sample alternative active site loop conformations, a monomer and the trimer of the Igni18 crystal structure were subjected to explicit-solvent, all-atom molecular dynamics (MD) simulations^35^ at 27 °C (only the monomer) and 90 °C (both trimer and monomer), the optimal growth temperature of *I. hospitalis*. At 27 °C, Igni18 exhibits lower mobility with a heavy-atom root mean square fluctuation (RMSF) mainly below 3 Å. The loop at the trimer interface (117–126, PVR5) exhibits the highest mobility in the monomer (Supplementary Fig. S5). At 90 °C, the mobility is higher only in certain regions, with the maximum of ~8 Å in the trimer interface loop (PVR5, Supplementary Fig. S5). This moderate increase is in line with the high thermostability of the enzyme (see below). With respect to the monomers at both temperatures, the trimer shows a lower mobility of the interface loop, as expected, but also, unexpectedly, a higher mobility of an active site loop (residues 54–59, Supplementary Fig. S5), which is significant (*p* < 0.05 for a two-sided *t*-test with *n* = 10) for residues 56, 58, and 59. Here, the interface loop having less space for movement in the trimer likely influences this active site loop with which it is directly interacting.

**Fig. 4:**
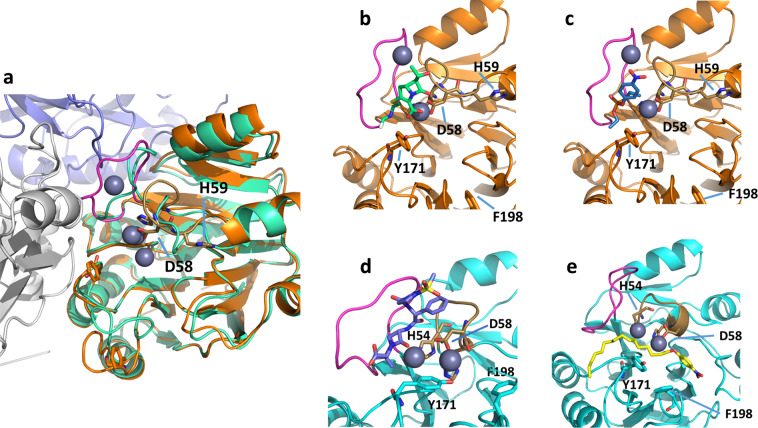
Predicted binding modes of substrates in MD-generated structures of the Igni18 monomer and trimer. **a** Differences between the X-ray crystal structure (green) and an exemplary MD-generated structure (orange, navy, white) with the trimer interface loop (magenta) widening the pocket in the latter structure to allow substrate access. The active site loop binding the zinc ions is shown sand colored and in stick representation. Exemplary binding mode models of imipenem (**b**; lime), paraoxon-ethyl (**c**; blue), mezlocillin (**d**; navy), and *p*NP-palmitate (**e**; yellow) in the Igni18 trimer at 90 °C (**b**, **c**; orange) and the Igni18 monomer at 27 °C (**d**, **e**; light blue). The interface loop is colored magenta while the active site loop binding the zinc ions is shown sand colored and in stick representation. Y171 and F198, which interact with e.g. *p*NP-palmitate are shown in stick representation and labeled.

Each MD trajectory was separately clustered with respect to symmetry corrected RMSD in order to obtain a high structural diversity of active site conformations for docking ligands using Autodock/DrugScore2018. To do so, the cluster representative of the respective largest cluster of each trajectory was used. For both the monomers and the trimer of Igni18, substrate binding poses that locate the cleaved bond close to the Zn^2+^ ions were identified using the cluster with the best scores, which also includes at least 20% of all poses (Fig. 4a–d). In all binding modes, the trimer interface loop has moved towards the trimer interface and away from the active center, compared to the crystal structure (Fig. 4e). This allows either the accommodation of a ligand between one Zn^2+^ ion and the trimer interface loop (PVR5, Fig. 4c, d) or the movement of a Zn^2+^ ion towards this loop, which opens a deeper pocket between the Zn^2+^ ions to accommodate hydrophobic ligands (Fig. 4a, b). An analysis with computed atlas of surface topography of proteins (CASTp^36^) of the active sites of the crystal structure and the 27 °C monomer structure show a solvent accessible surface area of in total 0 Å^2^ and 30.8 Å^2^, respectively, which explains the differences.

### Thermal stability, unfolding, and refolding

**Fig. 5:**
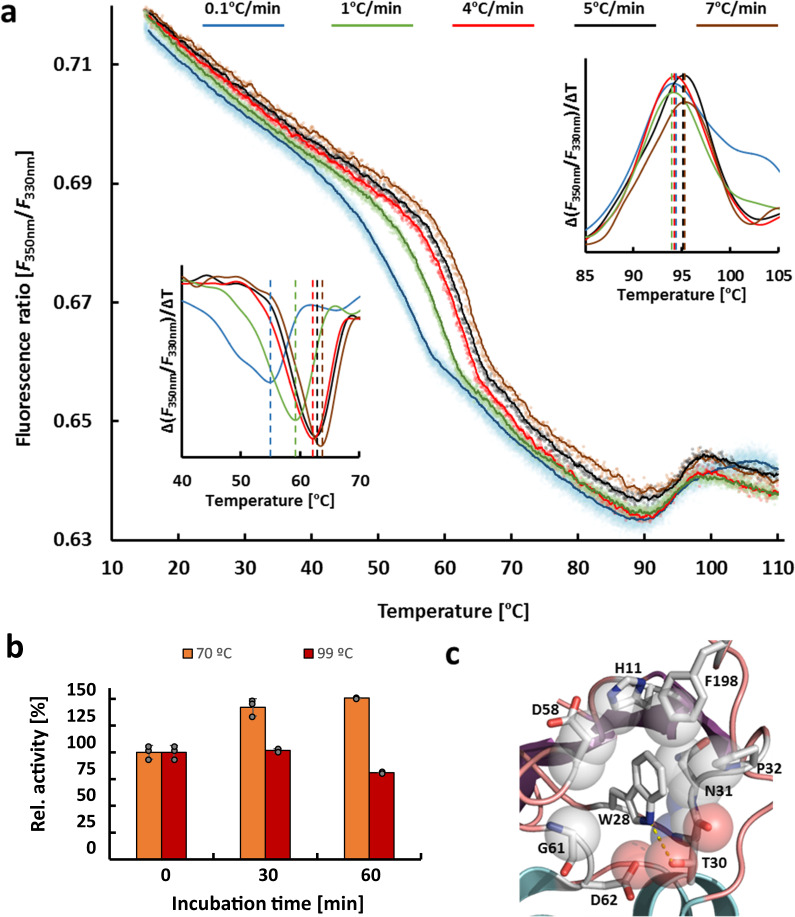
Two-step thermal unfolding pathway of Igni18. Effect of temperature heating rates on the thermal unfolding of native Igni18 analyzed by nanoDSF, measuring the intrinsic protein fluorescence from 15 °C to 110 °C (**a**). Incubation at 70 °C activates the protein whereas periods longer than 30 min at 99 °C are irreversible and lead to activity loss (**b**). Error bars represent the relative standard deviation (*n* = 3 independent experiments). The binding pocket of W28, used for the analysis, shows a strong hydrophobic character (**c**).

Igni18 is an extremely thermostable enzyme indicated by a half-life time of ~48 h at 90 °C (Supplementary Fig. S6). Thermal unfolding was measured by changes of intrinsic protein fluorescence upon heating by nanoDSF (nano differential scanning fluorimetry). Igni18 contains a single tryptophan residue (W28, Fig. 5c) partially exposed on the protein surface, but distant from the interface between the monomers, representing an ideal system for nanoDSF analysis (Supplementary Fig. S6). The thermal unfolding curve of Igni18 recorded between 15 °C and 110 °C shows two transitions, one in the range of ~55–65 °C and the second one at ~95 °C (Fig. 5a and Supplementary Data S2). The melting temperature for the low-temperature unfolding increased as the heating rates increased while the high-temperature unfolding transition did not show a heating rate dependency (Fig. 5a). Reversible unfolding of Igni18 was observed as the protein, which has been heated to 70 °C and then cooled, showed not only complete restoration of phosphodiesterase activity, but even a 50% increase (Fig. 5b and Supplementary Data S2). In contrast, the irreversibility of high-temperature unfolding was proposed from phosphodiesterase activity measurements (Fig. 5b). To verify this, refolding experiments were performed in which native Igni18 was heated up to 110 °C followed by cooling to 20 °C (identical heating and cooling rates). Igni18 regained fluorescence after refolding to reach a final fluorescence nearly identical to the initial fluorescence of the non-heated protein (Supplementary Fig. S6). Hence, heating of Igni18 to 110 °C does not lead to irreversible aggregation. These unfolding/refolding experiments and enzyme activity measurements indicate a two-step unfolding pathway for Igni18. Native Igni18 is partially unfolded at ~55–65 °C and is fully activated between 65 and 90 °C, temperatures at which *I. hospitalis* grows optimally^15^. Further heating of Igni18 above 95 °C results in inactive enzyme, which cannot be refolded by cooling, but is not prone to aggregation.

To find out if divalent metal cations have a structural role, Igni18 was incubated with EDTA in order to remove the cations before thermal unfolding measurements were conducted. The melting temperatures of metal-free Igni18 of 58.4 °C and 93.9 °C were nearly identical to those of native Igni18 (Supplementary Fig. S6). This indicates that metal cations do not stabilize the structure of Igni18.

## Discussion

Igni18 is highly promiscuous and thermostable and serves as a prototype to study the evolution of the remarkably diverse MβL superfamily. Its structure resembles the backbone of a broad variety of different specialized MβLs that share highly similar active site architectures. Thus, this study points out the influence of variable loop regions and the incorporation of different metal ions to the functional diversification of this group of metalloenzymes. This could provide clues to how the activity of proteins from this family, which only have a general function prediction, could be classified better. In order to systematically quantify and define PVRs prone to evolution from the ancient MβL-fold to the more specialized forms, we developed a scoring system. We were able to identify ten PVRs for the proteins analyzed and describe main structural differences leading to the modulation of activity and specificity. As an example, elongation of PVR2 and reduction of PVR5 and PVR9 seems to have occurred as an early adaptation for RNA hydrolysis, since they are common features of KH-CPSF1, RNase J, and tRNase Z (Fig. 1c). Modular addition of specialized PVRs to the Igni18 backbone should lead to chimeric enzymes with the expected functions and structures very similar to those shown in Fig. 2. While we believe the hereby described PVR-analysis is able to describe the structural evolution of one protein superfamily fold, some restrictions may apply when comparing other proteins with less conserved or robust core structures. In order to apply this method to further protein families, a higher RMSD cut-off value for and alignment position may be chosen to determine the start and end of a PVR.

The high structural similarity between the MβL from *T. maritima* (PDB 3X2Z) and Igni18 is accompanied by other parallels between the two organisms. *T. maritima* is a deep branching, hyperthermophilic bacterium. Its genome of 1.86 Mb is only 0.56 Mb larger than the *I. hospitalis* genome and up to 11% consist of archaeal genes^37^. Though the MβL 3X2Z is annotated as UlaG in *T. maritima*, other Ula-proteins (UlaA, UlaB, or UlaC) for l-ascorbate biosynthesis are missing like in *I. hospitalis* and there is no indication for this pathway in both organisms. Although there are structural similarities between Igni18 and the MβL UlaG, from e.g. *E. coli* and other Enterobacteria, there are also major differences. In contrast to Igni18 and most other MβLs, UlaG is depending on one Mn^2+^ ion and it is active in its hexameric form. It shows a side-activity against bis-*p*NPP^8^. The UlaG family represents a group that has specified its catalytic function to become a modern descendant of an ancient predecessor (Fig. 1c^7^).

The structure of Igni18 revealed two Zn^2+^ ions bound per monomeric unit. Igni18 can incorporate other ions as well and activity assays using 15 different substrates revealed a clear cofactor-dependency, except for lipase activity. As one of the most remarkable features, the activity of Igni18 appears to be switchable depending on the metal ions available. Metal-dependent activity changes could be a simple way to modulate the activity of this enzyme making it highly promiscuous. Indeed, different metal ions can greatly influence the reaction of enzymes like e.g. a β-lactamase from *Salmonella maltophilia* and a ribonuclease from *E. coli* on native and promiscuous substrates^38^. Therefore, Igni18 shows a metal-dependency typical for MβL superfamily members.

An extremely high binding affinity for Zn^2+^ was observed and a rarely occurring Zn^2+^ in the natural habitat^39^ will most likely be incorporated. Zn^2+^ and Cu^2+^ are known to have the highest binding affinity to metallo-proteins but most of these metal-binding preferences do not match the metal requirements of the respective proteins^40^. In the ICP-analysis, Cu^2+^ ions were not detected and *I. hospitalis* does not seem to have a Cu^2+^ transporter^20^, so the high activity on δ-dodecalactone can be considered as an artefact.

Next to lipase, lactonase and β-lactamase activities, Zn^2+^ contributes to Igni18´s promiscuity on phosphotriesters that do not occur naturally. It was shown recently, that only five mutations are necessary to evolve activity against methyl-parathion^41^. Phosphotriesterases could be used for detoxification of organophosphate-polluted environments.

Metal ions often play a crucial role in ligand binding and docking studies as they are part of the active center of e.g. metalloproteases and are targeted with specific metal-binding groups in ligands^42^. The knowledge-based scoring function DrugScore2018^33^, which was used in this study, explicitly considers the presence of metal ions. As a result, the predicted binding modes show that the functional groups in the docked ligands, which are cleaved by Igni18, are close to the Zn^2+^ ion so that it could attack these functional groups.

Altogether, the activity profile of Igni18 and the genomic analysis of *I. hospitalis* fit together in the so-called Patchwork Hypothesis^43^ claiming that ancestral organisms needed to function with a limited proteome and thus, enzymes with a broad specificity improved the metabolic capabilities^44,45^. Primitive, meaning relatively slow and unspecific, enzymes contributed to several different metabolic pathways as a possibility to compensate for the small genome. Typically, these enzymes have low turnover rates like Igni18. The evolution of new enzymatic functions can take place by gene duplication and subsequent functional divergence. The MβL-fold was already present in ancient predecessors from which modern catalytic activities have evolved^1,7^. Sequence- and structure-based analyses of the molecular evolution of UlaG show that these enzymes´ predecessors were RNA metabolizing enzymes (Fig. 1c left^8^). The genomic surrounding of *igni18* suggests this, too. Directly downstream of *igni18* is a predicted gene encoding a TatD-related DNase (ABU82429.1), a gene encoding PurL1 for purine biosynthesis (ABU82428.1) and an ATP-dependent DNA ligase (ABU82427.1). Still, it is important to mention that the promiscuous activities of Igni18 not necessarily need to play a physiological role. Even highly specific enzymes show side-activities without physiological relevance. Only when the reaction leads to a physiological advantage, generalist enzymes evolve to specialists^46^.

Nevertheless, these findings urge us to rethink annotations and metabolic pathway reconstruction. All-rounder enzymes from primordial organisms may catalyze reactions for different rudimentary and non-optimized pathways and some steps might even run coincidentally.

## Methods

### Cloning of *igni18* into *Escherichia coli* and *Pichia pastoris* as heterologous host

Amplification and cloning of the metallo-hydrolase gene *igni18* (NCBI accession number IGNI_RS06455; start nucleotide No. 1115579, end nucleotide No. 1114878^18^) into the pPICZ-A plasmid was accomplished by restriction cloning (*Eco*RI and *Not*I) in frame with the C-terminal Myc epitope and a hexahistidine tag. Transformation of *P. pastoris* X-33 cells with the resulting construct was performed according to the manual (EasySelect Pichia Expression Kit, Thermo Fisher Scientific, Waltham, MA, USA)^21^.

### Recombinant enzyme production in *P. pastoris* X33 (Mut^S^) and purification

Protein production was performed at 30 °C in Buffered extra-YNB (Yeast Nitrogen Base) Glycerol Methanol (BYGM) autoinduction medium^47^ for 46 h as described^21^, but the fermentation process was up-scaled to 10 L in an Infors HT Labfors benchtop bioreactor (13 L vessel volume, Infors AG, Bottmingen, Switzerland). The fermentation broth was concentrated by filtration (0.2 µm, Centramate™ 500 S Tangential Flow Filtration System, Pall, Dreieich, Germany) and centrifuged for 10 min at 10,000 rpm (F12-6×500 LEX, Sorvall RC6 Plus, Thermo Scientific, Massachusetts, USA). The pellet was stored at −80 °C until further use.

For purification of the His-tagged protein, 15.4 g of pellet corresponding to 1 liter of culture were thawed, lysed and purified by Ni^2+^-ion affinity chromatography as described before^21^. The eluted protein was concentrated and metal-ions were removed by stripping with 25 mM HEPPES buffer pH 7.5 containing 20 mM EDTA in an ultrafiltration unit (Vivaspin 20, Sartorius AG, Göttingen, Germany). The protein was subsequently dialyzed against 0.1 M potassium phosphate buffer or 5 mM EPPS pH 7, sterile filtered (0.2 µm) and stored in aliquots at 4 °C for up to several months. Protein purity was determined with SDS-PAGE and protein concentration was measured both colorimetrically with Bradford reagent at 595 nm and by UV-Vis-spectrometer at 280 nm using the specific protein extinction coefficient 17,420 M^−1^cm^−1^ (https://web.expasy.org/protparam). The concentration was adjusted to 1 or 0.1 mg mL^−1^ prior to use.

### Crystallization, structure determination, and cofactor analysis

Igni18 was crystallized in 0.3 M Mg(NO_3_)_2_ · 6 H_2_O, 0.1 M Tris pH 8, 22% (w/v) PEG 8000 at 20 °C via sitting-drop vapor diffusion and crystallographic data sets were collected from 2,500 frames at 0.05 ° rotation^21^. The structure of Igni18 containing six C-terminal histidine residues was solved by using the anomalous signal derived from the two bound Zn^2+^-ions. Here, we used the programs XSCALE^48^ and HKL2MAP^49^ to identify the Zn^2+^ site and to phase the initial model.

The crystal contained 1 molecule per asymmetric unit^3^, with a Matthews coefficient of 2.2 Å^3^ per Da. Iterative cycles of manual building were conducted in COOT^50^ with crystallographic refinement in REFMAC5^51^. The last rounds of refinement were done without restraints and with the individual, isotropic B-factors^50^ with crystallographic refinement in REFMAC5 with 93.5 % of the residues being in the favored region of the Ramachandran plot. Data collection and refinement statistics are given in Table 1. The PyMOL 2.3 and UCSF Chimera 1.14rc softwares were used for structural alignment, analysis, secondary structure assignment and visualization of protein structures (www.pymol.org, www.cgl.ucsf.edu/chimera).

A 12 mg mL^−1^ non-stripped protein solution in 50 mM Tris-HCl pH 8 was sent to Analytik Labor Schirmacher GmbH (Hamburg, Germany) for inductively coupled plasma mass spectrometry (ICP-MS) analysis. Among others, the presence of the divalent metals Mn, Fe, Co, Ni, Cu, and Zn was quantified. A buffer control was included as a blank.

### Sequence and structural alignments

A BLAST search against the non-redundant databank was performed in order to examine the occurrence of Igni18 homologs within different phylogenetic groups. Multiple sequence alignments were performed with M-Coffee from the T-Coffee software package^52,53^ and phylogenetic analysis was conducted with MEGA X^54^.

The isolated monomer structure of Igni18 was uploaded to the mTM-align^55^ and DALI^56^ servers for a search against the Protein Data Bank (PDB) to detect structural similarities with other proteins that are otherwise not detectable by sequence comparisons.

### Enzyme activity assays for substrate promiscuity and temperature/pH optimum

#### β-lactamase assay

The β-lactamase activity of Igni18 was assayed against the chromogenic substrate Nitrocefin (Merck, Darmstadt, Germany) in phosphate buffer pH 7 at 40 °C. 10 mM stock solutions were prepared in dimethyl sulfoxide (DMSO) and stored at −20 °C. Standard assays were performed in 200 µL containing 190 µL buffer with 0.5 mM substrate and 10 µL enzyme solution (1 mg mL^−1^). Degradation of the substrate was monitored by measuring the absorbance at 490 nm on a Biotek Synergy HT (Bad Friedrichshall, Germany) plate-reader. All assays were performed in triplicate and a buffer control was added. An extinction coefficient of 16,000 M^−1^cm^−1^ was used for kinetic calculations. Activity on other β-lactams was indirectly assayed via a disc-diffusion antibiotic susceptibility test. Mezlocillin 30 µg (MEZ 30), imipenem 10 µg (IPM 10), cefamandole 30 µg (MA 30), loracarbef 30 µg (LOR 30), cefaclor 30 µg (CEC 30), cefotaxime 30 µg (CTX 30), and cefotiam 30 µg (CFT 30) susceptibility disks (Thermo Fischer Scientific, Waltham, MA, USA) were incubated overnight at 40 °C with 30 µL 0.1 M potassium phosphate buffer pH 8 containing 1 mg mL^−1^ metal-free Igni18 and 1 mM NiCl_2_ or ZnCl_2_. A control without enzyme was included. The antibiotic susceptibility test was carried out using these pre-incubated disks the next day. They were laid on LB-agar on which *E. coli* DH5α cells had been plated out. After incubation overnight at 37 °C, the zone of inhibition (ZOI) around each disc was determined for every antibiotic and condition. The reduction of the ZOI was expressed in percentage.

#### Lactonase assay

Hydrolysis of γ-dodecalactone and δ-dodecalactone (Sigma-Aldrich, Munich, Germany) releases a proton under physiological conditions. Due to the volatile nature of the substrates, activity was assayed at 40 °C in 5 mM EPPS buffer pH 8 with 0.45 mM phenol red. 100 mg mL^−1^ stock solutions were prepared in dimethyl sulfoxide (DMSO) and stored at -20 °C. 10 µL of substrate and 5 µL protein (1 mg mL^−1^) were added to 235 µL buffer with phenol red and absorbance at 550 was monitored overnight. All assays were performed in triplicate and a buffer control was added. A standard curve to correlate the measured values to the release of carboxylic acids was performed with acetic acid.

#### Activity assays with *p*NP substrates

*p*NP-carboxyl esters in various acyl chain lengths (*p*NP-C2 to C18), *p*NP-phosphate (*p*NPP), bis-*p*NP-phosphate (bis*-p*NPP), paraoxon-ethyl and parathion-ethyl were purchased from Sigma-Aldrich (Munich, Germany), *p*NP-phenylphosphonate (*p*NPPP), and *p*NP-phosphorylcholine (*p*NPPC) from Biomol GmbH (Hamburg, Germany). In all, 10 mM stock solutions were prepared in 2-propanol and stored at −20 °C. Standard assays were performed in 200 µL containing 190 µL buffer with 1 mM substrate and 10 µL enzyme solution (0.1 or 1 mg mL^−1^) and incubated at 90 °C for 30 min (unless otherwise indicated). The effect of different divalent metals (Mg, Ca, Mn, Fe, Co, Ni, Cu, and Zn) on the enzyme activity was studied by adding 1 mM of the corresponding metal chloride salts to the reaction. Reactions were stopped by the addition of 20 µL 2 M Na_2_CO_3_ and the formation of *p*-nitrophenolate was measured spectrophotometrically at 405 nm on a Biotek Synergy HT (Bad Friedrichshall, Germany) plate-reader. All assays were performed in triplicate and a buffer control was added. A standard curve with known concentrations of pure *p*-nitrophenolate was used to determine the extinction coefficient (ε) of the hydrolysis product. Temperature optimum was determined in the range of 40–95 °C. The optimal pH was assayed with different buffers between pH 4 and 10 (0.1 M; pH 4–6: citrate-phosphate buffer; pH 7–8: tris buffer; pH 9–10: carbonate-bicarbonate buffer). For kinetic studies, several substrate concentrations were assayed and aliquots were taken at different time points of the reaction and stored on ice until absorbance was measured. One unit (U) was defined as the amount of protein converting 1 µmol substrate per minute. *V*_max_, *K*_m_, and *k*_cat_ were calculated according to Michaelis–Menten kinetics.

### Molecular dynamics simulations

The monomer and trimer of the X-ray crystal structure were subjected to all-atom MD simulations. The variants were protonated with PROPKA^57^ according to pH 8, neutralized by adding counter ions, and solvated in an octahedral box of TIP3P^58^ water with a minimal water shell of 12 Å around the solute. The Amber package of molecular simulation software^59^ and the ff14SB force field^60^ were used to perform the MD simulations. The Zn^2+^ ions were treated with the Li-Merz^61^ parameters. To cope with long-range interactions, the Particle Mesh Ewald method^62^ was used; the SHAKE algorithm^63^ was applied to bonds involving hydrogen atoms. As hydrogen mass repartitioning^64^ was utilized, the time step for all MD simulations was 4 fs with a direct-space, non-bonded cut-off of 8 Å. At the beginning, 17,500 steps of steepest decent and conjugate gradient minimization were performed; during 2500, 10,000, and 5000 steps positional harmonic restraints with force constants of 25 kcal mol^−1^ Å^−2^, 5 kcal mol^−1^ Å^−2^, and zero, respectively, were applied to the solute atoms. Thereafter, 50 ps of NVT (constant number of particles, volume, and temperature) MD simulations were conducted to heat up the system to 100 K, followed by 300 ps of NPT (constant number of particles, pressure, and temperature) MD simulations to adjust the density of the simulation box to a pressure of 1 atm and to heat the system to 300 K or 363 K. During these steps, a harmonic potential with a force constant of 10 kcal mol^−1^ Å^−2^ was applied to the solute atoms. As the final step in thermalization, 300 ps of NVT-MD simulations were performed while gradually reducing the restraint forces on the solute atoms to zero within the first 100 ps of this step. Afterwards, for 300 K and 363 K each, ten independent production runs of NVT-MD simulations with 2000 ns length each were performed. For this, the starting temperatures of the MD simulations at the beginning of the thermalization were varied by a fraction of a Kelvin. The location of the binding pocket was tracked using VMD^65^. Conformations of all the ten independent production runs for each variant for both temperatures were subsequently clustered individually by a hierarchical agglomerative clustering algorithm as implemented in CPPTRAJ^66^ using the root-mean-square deviation of heavy atoms after superimposition to all residues as a measure and aiming for five clusters. The cluster representative of the largest cluster for each variant was then used for docking.

### Molecular docking

For the molecular docking, ligands imipenem (IPM), mezlocillin (MEZ), paraoxon-ethyl, and *p*NP-palmitate (*p*NP-C16) were drawn and converted into a 3D structure with Maestro (www.schrodinger.com/maestro). The ligands were subsequently docked into the binding pocket of the respective crystal structure or cluster representative utilizing a combination of AutoDock^32^ as a docking engine and the DrugScore2018^33^ distance-dependent pair-potentials as an objective function. In the docking, default parameters were used, with the exception of the clustering RMSD cutoff, which was set to 2.0 Å. Binding modes were considered valid, if they were located in the binding pocket and contained in the largest cluster, which comprised at least 20 % of all docking poses.

### Thermal stability, unfolding, and refolding

Initial enzyme stability assays were conducted by incubating the purified, Zn^2+^-containing enzyme at 90 °C. Between an incubation time of 15 min and 144 h (6 days), samples were taken at certain time points from the vials in triplicates and stored at 8 °C. The residual activity was subsequently measured with *p*NP-myristate (C14) at 90 °C.

Unfolding and refolding of native Igni18 were studied by measuring the intrinsic protein fluorescence at 330 nm and 350 nm using a Prometheus nanoDSF (NanoTemper, Munich, Germany) device. The protein samples loaded into the measuring capillaries (Prometheus NT.Plex nanoDSF Grade Standard Capillary Chips) were heated from 15 °C to 110 °C followed by cooling from 110 °C to 15 °C at rates of 0.1 °C, 1 °C, 4 °C, 5 °C, and 7 °C min^−1^. The ratio of *F*_350 nm_ and *F*_330 nm_ and its first derivative were calculated with the PR.ThermControl software provided by the company.

### Statistics and reproducibility

All experiments reported in this study have been reproduced and similar results have been obtained in at least three independent biological repeats. A two-sided *t*-test with *n* = 10 was performed for giving a significant *p*-value.

### Reporting summary

Further information on research design is available in the Nature Research Reporting Summary linked to this article.

## Supplementary information

Supplementary Information

Description of Supplementary Files

Supplementary Data 1

Supplementary Data 2

Reporting Summary

## Data Availability

The data that support the findings of this study are available from the corresponding author upon reasonable request. The Igni18 coordinates can be accessed in the PDB (6HRG). Results of the structural searches performed with mTM-align and the DALI servers as well as data underlying Figs. 3 and 5a, b are available within Supplementary Data S1 and Supplementary Data S2.
