# Supplementary material for: A promiscuous ancestral enzyme´s structure unveils protein variable regions of the highly diverse metallo-β-lactamase family
Source: Commun Biol. 2021 Jan 29;4:132. doi: 10.1038/s42003-021-01671-8 (PMC7846560; doi:10.1038/s42003-021-01671-8)
Supplement: Supplementary file 1 — Supplementary Information [file 42003_2021_1671_MOESM1_ESM.pdf]

A promiscuous ancestral enzyme´s structure unveils protein variable regions of the highly diverse metallo-β-lactamase family

Pablo Perez-Garcia[1], Stefanie Kobus[2], Christoph G. W. Gertzen[2], Astrid Hoeppner[2], Nicholas Holzscheck[1], Christoph Heinrich Strunk[3], Harald Huber[4], Karl-Erich Jaeger[3,5], Holger Gohlke[6,7], Filip Kovacic[3], Sander H. J. Smits[2,8], Wolfgang R. Streit[1], Jennifer Chow[1]*

[1] Department of Microbiology and Biotechnology, University of Hamburg, Ohnhorststrasse 18, 22609 Hamburg, Germany
[2] Center for Structural Studies (CSS), Heinrich Heine University Düsseldorf, Universitätsstrasse 1, 40225 Düsseldorf, Germany
[3] Institute of Molecular Enzyme Technology (IMET), Heinrich Heine University Düsseldorf, 52426 Jülich, Germany
[4] Institute for Microbiology and Archaeal Center, Regensburg University, 93035 Regensburg, Germany
[5] Institute of Bio- and Geosciences IBG-1: Biotechnology, Forschungszentrum Jülich GmbH, 52426 Jülich, Germany
[6] John von Neumann Institute for Computing (NIC), Jülich Supercomputing Centre (JSC) & Institute of Biological Information Processing (IBI-7: Structural Biochemistry), Forschungszentrum Jülich GmbH, 52425 Jülich, Germany
[7] Institute for Pharmaceutical and Medicinal Chemistry, Heinrich Heine University Düsseldorf, 40225 Düsseldorf, Germany
[8] Institute of Biochemistry, Heinrich Heine University Düsseldorf, 40225 Düsseldorf, Germany.

*Corresponding author: Jennifer Chow (jennifer.chow@uni-hamburg.de)

Supplementary Materials

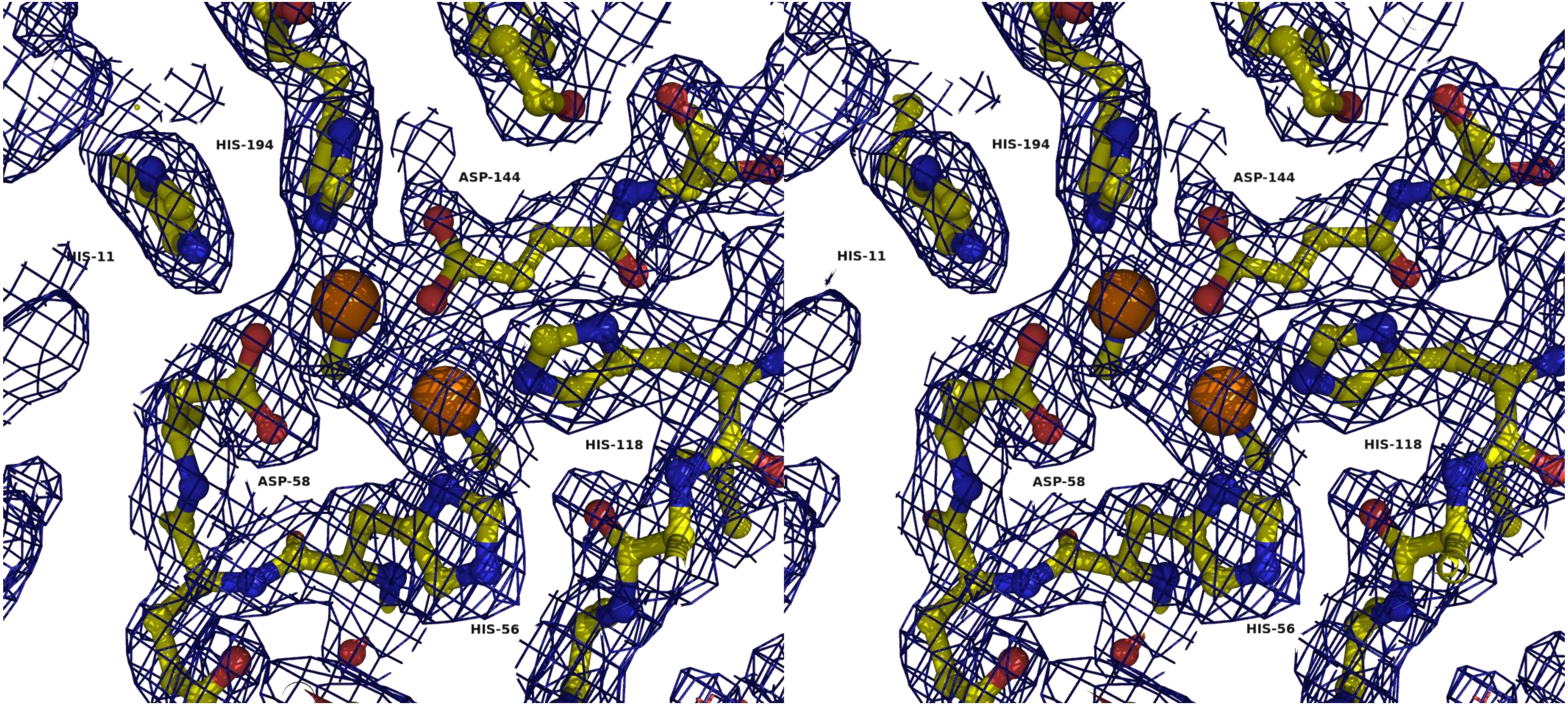

Supplementary Figure S1: Stereo image of the Zn^{2+} binding site of Igni18. Electron density is shown at 1.0 sigma. Amino acids involved in the binding of Zn^{2+} are labeled. Yellow: carbon atoms; red: oxygen atoms; blue: nitrogen atoms. Zn^{2+} is shown in orange spheres.

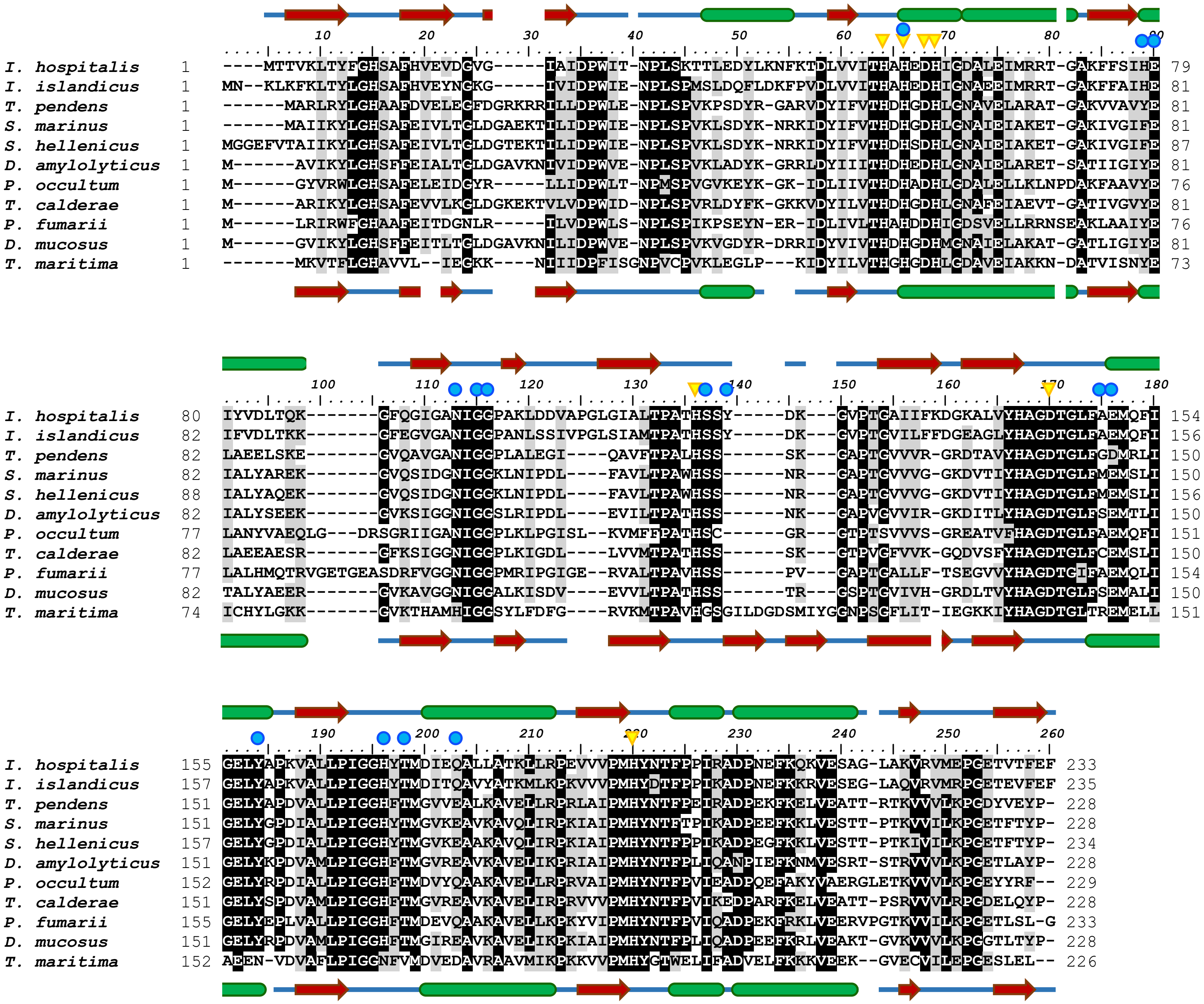

Supplementary Figure S2: Sequence comparison between Igni18 and its homologs. Nine homologs were selected for sequence comparison. Each sequence is labeled by the species name: *Ignicoccus hospitalis* (Igni18; WP_012123394.1); *Ignicoccus islandicus* (WP_075049418.1); *Thermofilum pendens* (WP_011753155.1); *Staphylothermus marinus* (WP_011839182.1); *Staphylothermus hellenicus* (WP_052833686.1); *Desulfurococcus amylolyticus* (WP_042667863.1); *Pyrodictium occultum* (WP_058370634.1); *Thermogladius calderae* (WP_048162871.1); *Pyrolobus fumarii* (WP_014026484.1); *Desulfurococcus mucosus* (WP_013562750.1). WP_004080208.1 (*Thermotoga maritima*) is also included in the analysis. Black shading represents conservation of more than 80%, grey shading highlights similar amino acid residues. The secondary structures derived from Igni18 and the metallo-hydrolase from *T. maritima* are shown respectively above and below its sequence (α-helixes in green, β-sheets in red). The seven amino-acid residues necessary for metal coordination are marked with a yellow inverted triangle (∇); the ones needed for trimerization are highlighted with a blue circle (○).

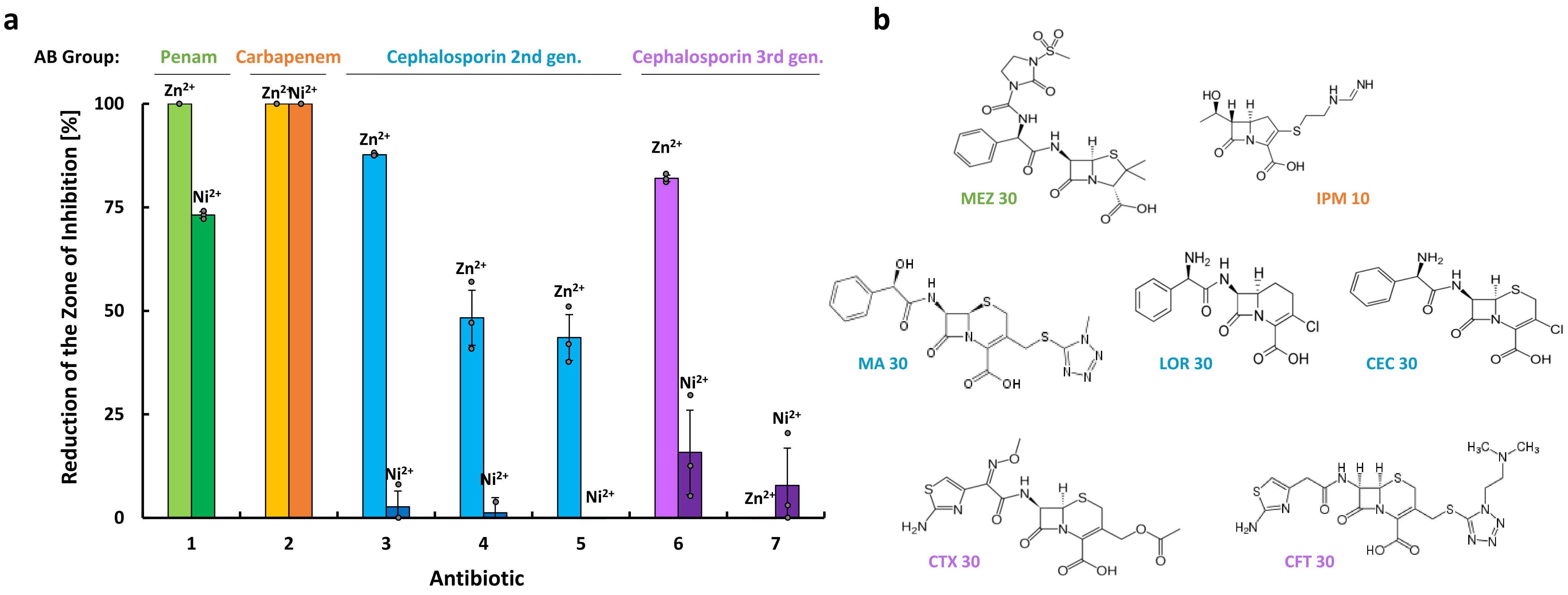

Supplementary Figure S3: The β-lactamase activity of Igni18 on seven different antibiotics from four different groups is metal-dependent. Disc-diffusion assay shows activity of Igni18 on seven different β-lactam antibiotics. Error bars represent the relative standard deviation (n=3 independent experiments). Molecular structures of the seven different antibiotic compounds (b).

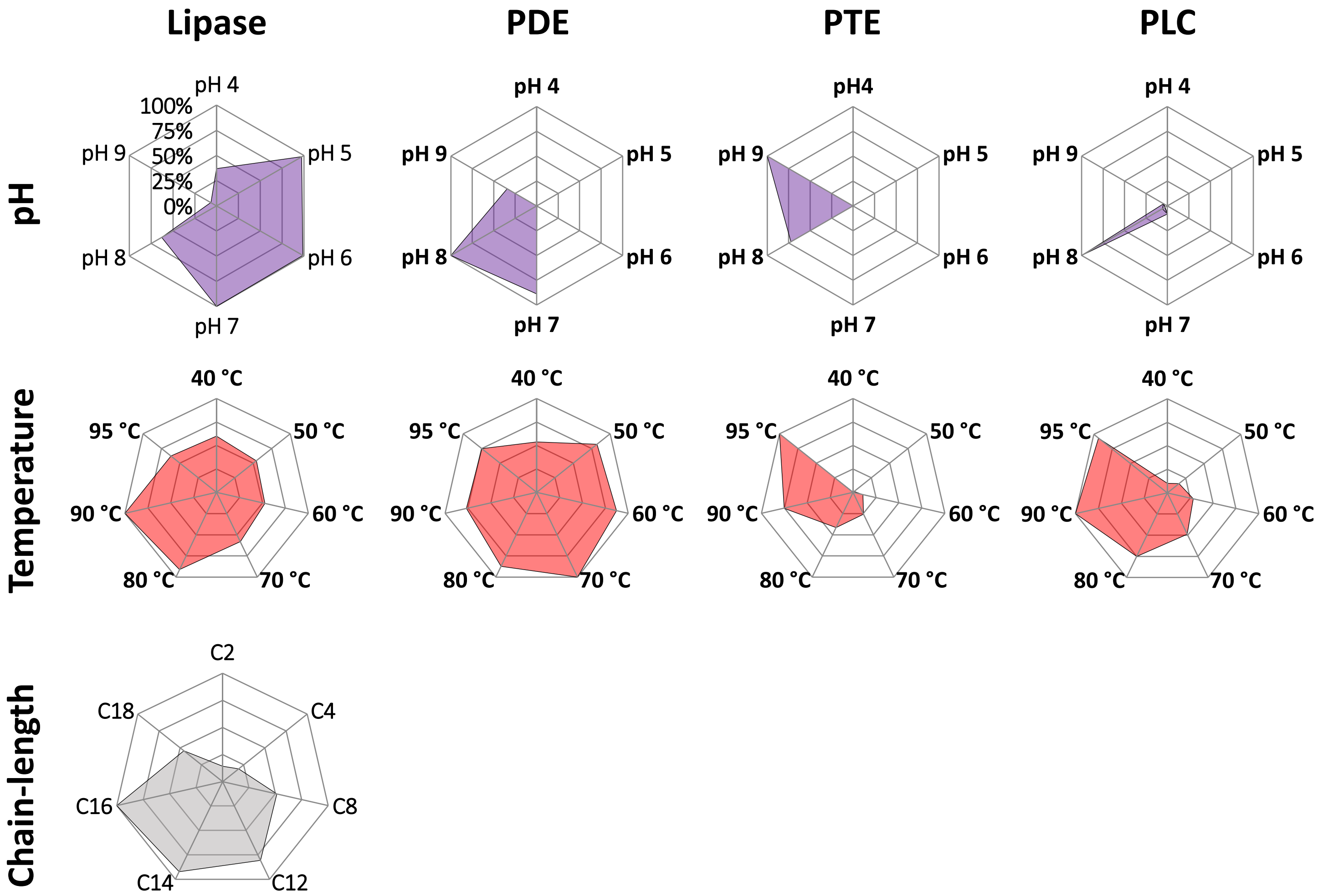

Supplementary Figure S4: Determination of optimal parameters for diverse activities of Igni18. Lipase activity temperature and pH optimum were determined on *p*NP-palmitate (*p*NP-C16), phosphodiesterase (PDE) on bis-*p*NPP, phosphotriesterase (PTE) on paraoxon and phospholipase C (PLC) on *p*NP-PC. For lipase activity, optimal acyl-chain length was determined to be 16 C-atoms.

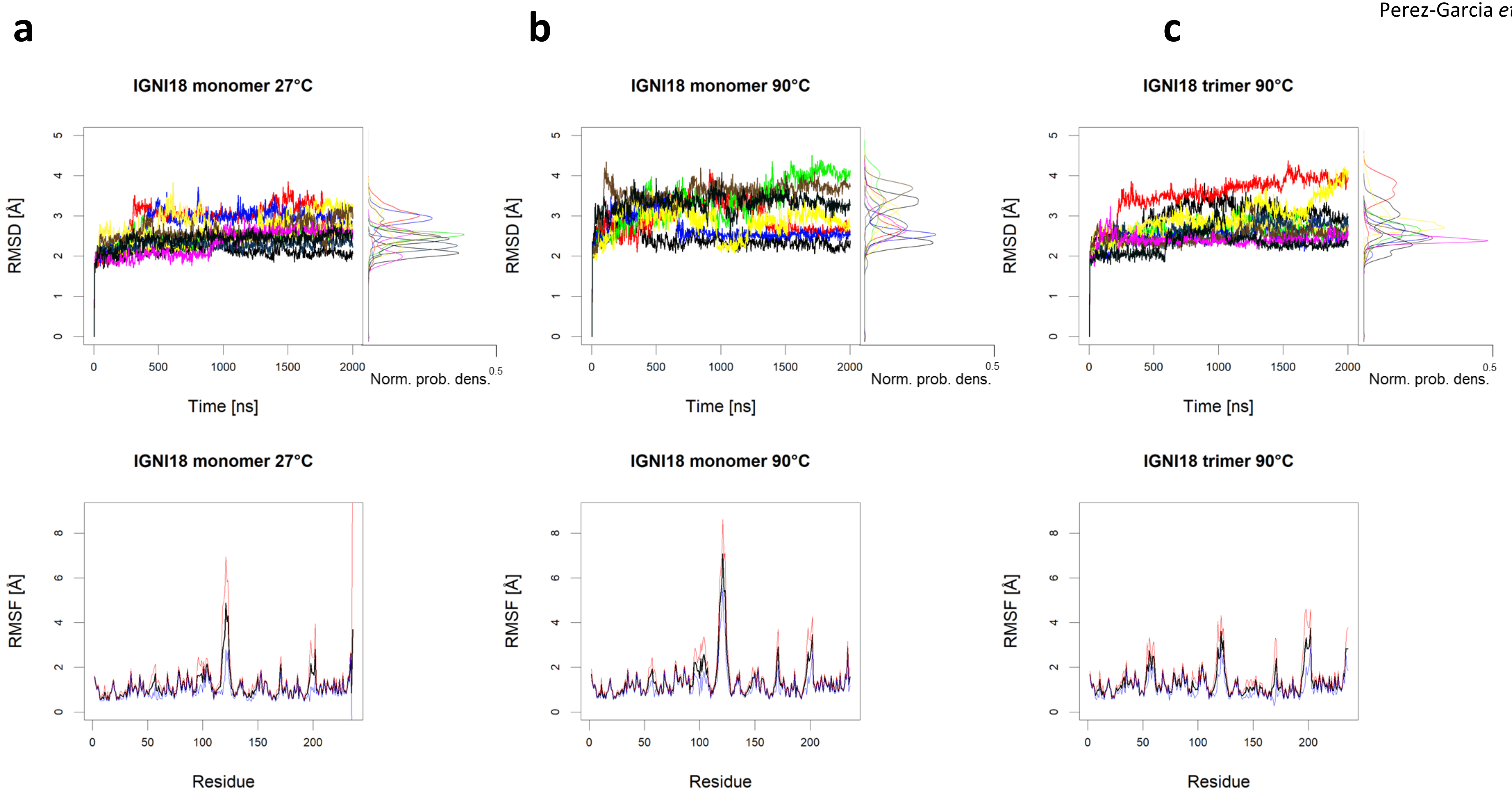

Supplementary Figure S5: Root mean square deviations (RMSD) and fluctuations (RMSF). The heavy atom RMSD (up) and RMSF (down) of the Igni18 monomer at 27 °C (a), the Igni18 monomer at 90 °C (b), and the Igni18 trimer at 90 °C (c) after superimposing to the crystal structure, respectively. The trimer was calculated as the single monomers. The different coloured lines in the RMSD plots represent the ten different replicas. The density distribution function is shown on the right side of the RMSD plots and the area under the curve is normalized to 1. The RMSD is shown as the mean (black) plus (red) and minus (blue) the standard deviation (n=10 independent experiments).

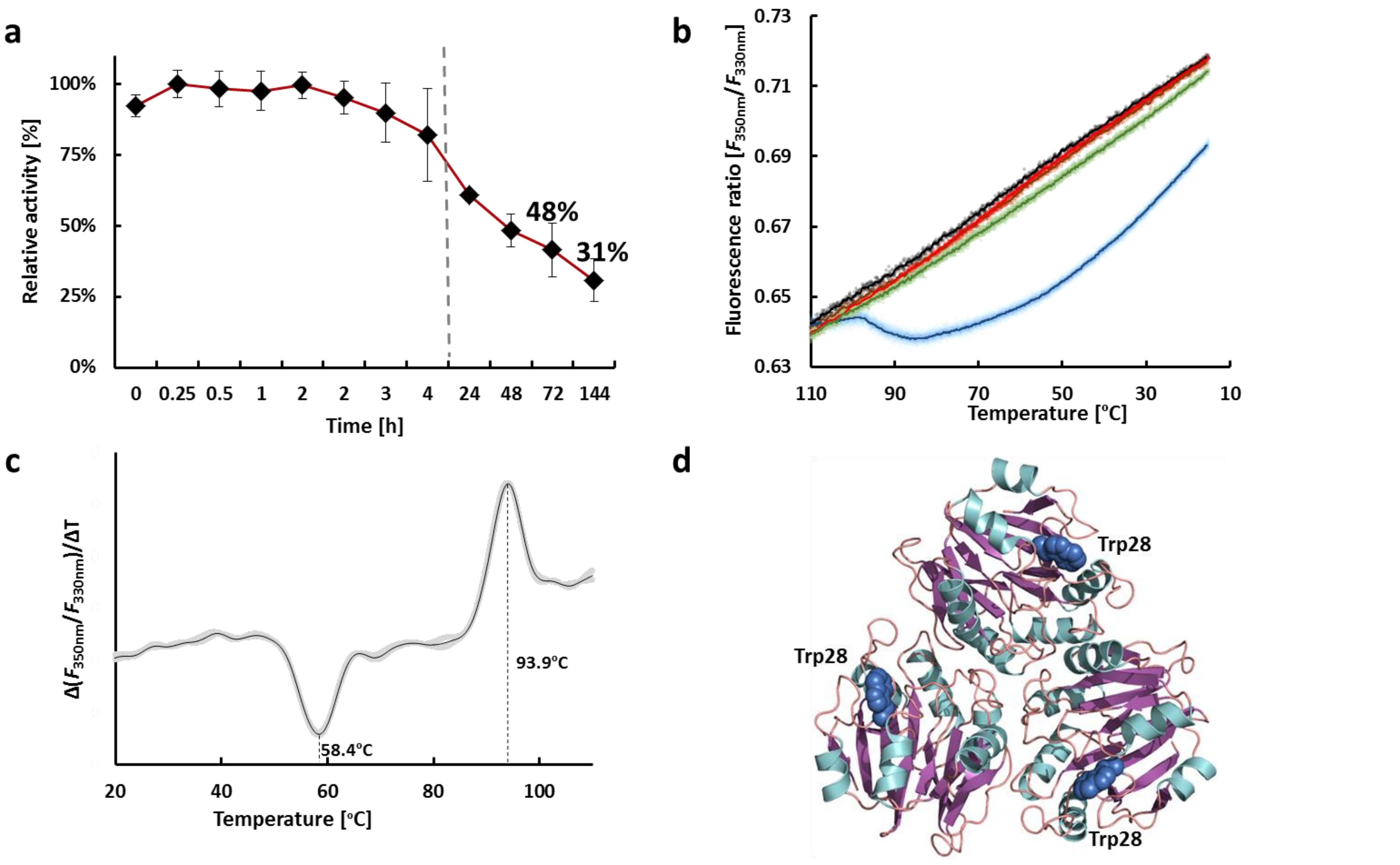

Supplementary Figure S6: Thermal stability of Igni18. The protein exhibited a half-life of 48 h at 90°C (a; assayed on *p*NP-C14). Error bars represent the relative standard deviation (n=3 independent experiments). Thermal refolding of Igni18 (b). Thermal unfolding of Igni18 in the presence of EDTA shows that metal cations do not have a heat-stabilizing role (c). Location of the three W28 used for the nanoDSF analysis within the trimeric structure of Igni18 (d).

Supplementary Table S1: Identification of the Protein Variable Regions of enzymes from the MβL family.

ENZYME	PDB	PVR1			PVR2			PVR3			PVR4			PVR5			PVR6			PVR7			PVR8			PVR9			PVR10		
		start	end	length	start	end	length	start	end	length	start	end	length	start	end	length	start	end	length	start	end	length	start	end	length	start	end	length	start	end	length
Igni18	6HRG	1	K 5	5	H 11	A 13	3	W 28	T 47	20	I 77	G 91	15	T 117	P 126	10	F 132	L 138	7	L 147	P 160	14	G 168	M 173	6	Y 195	V 220	26	T 230	K 234	5
MβL	3X2Z	1	K 2	2	H 8	V 10	3	F 24	I 41	18	N 71	T 85	15	V 107	P 124	18	T 130	K 135	6	L 144	V 156	13	G 164	M 169	6	Y 191	C 215	25	E 225	L 226	2
ITCase	6BRM	1	K 2	2	N 8	T 10	3	M 25	V 60	36	Q 90	I 106	17	Q 127	A 146	20	T 152	T 158	7	W 167	P 180	14	G 188	M 200	13	M 222	I 248	27	A 258	TRP 262	5
β-hydroxylase	4JO0	1	V 252	252	H 258	C 260	3	V 275	I 298	24	P 329	V 353	25	L 375	K 386	12	R 392	S 397	6	N 406	V 422	17	E 430	S 461	32	M 483	A 521	39	R 531	LEU 532	2
NAPE-PLD	4QN9	1	R 126	126	H 132	T 134	3	I 149	P 177	29	P 212	V 227	16	Q 252	W 267	16	L 273	R 278	6	Y 287	F 301	15	G 309	V 322	14	W 344	F 375	32	Y 385	ASP 389	5
KH-CPSF1	2XR1	1	R 182	182	G 188	C 196	9	G 211	I 235	25	T 266	T 305	40	G 328	S 333	6	H 339	N 346	8	Y 355	V 371	17	T 379	S 581	203	G 605	T 625	21	R 635	LEU 637	3
RNASE J	5HAA	1	G 5	5	G 11	M 19	9	G 34	V 75	42	T 105	I 128	24	Q 151	T 157	7	H 163	A 168	6	F 177	V 200	24	T 208	A 389	182	G 411	L 434	24	Y 444	GLU 447	4
tRNase Z	4GCW	1	E 2	2	T 8	S 21	14	G 39	I 56	18	P 93	I 117	25	I 139	A 144	6	Q 150	S 205	56	V 214	C 225	12	T 233	S 248	16	I 270	S 293	24	N 303	GLY 307	5
UlaG	2WYM	1	A 39	39	C 45	G 47	3	W 63	I 110	48	P 144	C 161	18	F 183	A 209	27	K 215	S 220	6	Y 229	I 242	14	G 250	M 260	11	H 282	P 312	31	T 322	PHE 338	17
Unknown funct.	3BV6	1	S 39	39	C 45	G 47	3	W 63	I 110	48	P 144	C 161	18	D 184	A 210	27	E 216	S 221	6	Y 230	I 243	14	G 251	M 261	11	H 283	P 313	31	T 323	LEU 355	33
**RMSD [Å]**		**---**	**1.64**		**1.651**	**1.251**		**1.907**	**1.801**		**1.498**	**1.918**		**1.572**	**1.43**		**1.659**	**1.417**		**1.738**	**1.296**		**1.979**	**1.9**		**1.574**	**1.959**		**1.356**	**---**	

Supplementary Table S2: Summary of the enzymatic activities of Igni18 on the different substrates.

EC	Substrate	Structure	Metal ion*	Activity [U/mg]
EC 3.1.1.1/3 Esterase / Lipase	*p* NP-caprylate (C8)	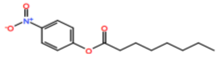	n.d.	n.d.
	p NP-laurate (C12)	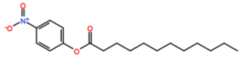	n.d.	n.d.
	p NP-myristate (C14)	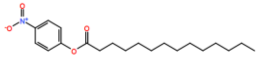	n.d.	n.d.
	p NP-palmitate (C16)	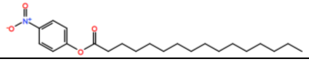	No influence but Ni^{2+} decreases activity	0.004
EC 3.1.4 PDE	Bis(*p* NP)-phosphate	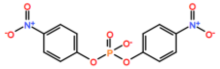	Ni^{2+}	1.26
	p NP-phenylphosphonate	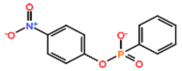	Ni^{2+} (Mn^{2+}, Co^{2+})	1.72
EC 3.1.8.1 PTE	Paraoxon-ethyl	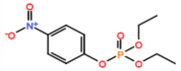	Zn^{2+}	0.072
	Parathion-ethyl	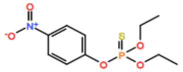	Zn^{2+} (Co^{2+}, Mg^{2+}, Ni^{2+})	0.033
EC 3.1.4.3 PLC	*p* NP-phosphorylcholine	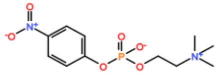	Ni^{2+} (Mn^{2+})	0.45
EC 3.5.2.6 β-lactamase	Nitrocefin	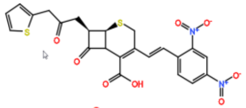	Cu^{2+}, Zn^{2+}, Ni^{2+}	0.009
	Mezlocillin (Penicillin)	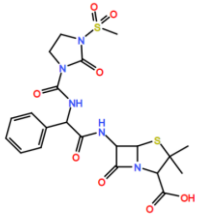	Zn^{2+} (Ni^{2+})	See inhibition assay
	Imipenem (Carbapenem)	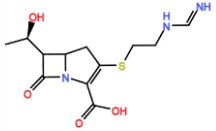	Zn^{2+}, Ni^{2+}	See inhibition assay
	Cephalosporines**	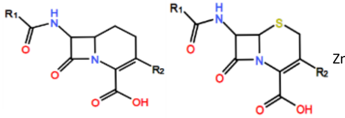	Zn^{2+} (Ni^{2+})	See inhibition assay
EC 3.1.1.25 Lactonase	γ-Dodecalactone	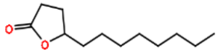	Cu^{2+}	0.233
	δ-Dodecalactone	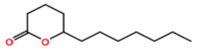	Cu^{2+}	4.56

*Metal ion leading to the highest activity, other ions leading to a lower activity are written in braquets;
PDE: Phosphodiesterase; **PTE:** Phosphotriesterase,
PLC: Phospholipase C;
****Cephalosporines used:** Loracarbef (LOR), Cefotiam (CFT), Cefamandole (MA), Cefotaxim (CTX), Cefaclor (CEC).
N.d.; not determined.
