# Supplementary material for: A promiscuous ancestral enzyme´s structure unveils protein variable regions of the highly diverse metallo-β-lactamase family
Source: Commun Biol. 2021 Jan 29;4:132. doi: 10.1038/s42003-021-01671-8 (PMC7846560; doi:10.1038/s42003-021-01671-8)
Supplement: Supplementary file 2 — Description of Supplementary Files [file 42003_2021_1671_MOESM2_ESM.pdf]

Description of Additional Supplementary Files

File name: Supplementary Data S1

Description: Results of the structural searches performed with mTM-align and the DALI servers.

File name: Supplementary Data S2

Description: Data underlying Figure 3 and Figure 5a and b.
