# Supplementary material for: A promiscuous ancestral enzyme´s structure unveils protein variable regions of the highly diverse metallo-β-lactamase family
Source: Commun Biol. 2021 Jan 29;4:132. doi: 10.1038/s42003-021-01671-8 (PMC7846560; doi:10.1038/s42003-021-01671-8)
Supplement: Supplementary file 5 — Reporting Summary [file 42003_2021_1671_MOESM5_ESM.pdf]

nature research

Corresponding author(s): Jennifer Chow

Last updated by author(s): Dec 12, 2020

Reporting Summary

Nature Research wishes to improve the reproducibility of the work that we publish. This form provides structure for consistency and transparency in reporting. For further information on Nature Research policies, see our Editorial Policies and the Editorial Policy Checklist.

Statistics

For all statistical analyses, confirm that the following items are present in the figure legend, table legend, main text, or Methods section.

n/a	Confirmed	
☐	☒	The exact sample size (*n*) for each experimental group/condition, given as a discrete number and unit of measurement
☐	☒	A statement on whether measurements were taken from distinct samples or whether the same sample was measured repeatedly
☐	☒	The statistical test(s) used AND whether they are one- or two-sided<br>*Only common tests should be described solely by name; describe more complex techniques in the Methods section.*
☒	☐	A description of all covariates tested
☒	☐	A description of any assumptions or corrections, such as tests of normality and adjustment for multiple comparisons
☐	☒	A full description of the statistical parameters including central tendency (e.g. means) or other basic estimates (e.g. regression coefficient) AND variation (e.g. standard deviation) or associated estimates of uncertainty (e.g. confidence intervals)
☒	☐	For null hypothesis testing, the test statistic (e.g. F, t, r) with confidence intervals, effect sizes, degrees of freedom and P value noted<br>*Give P values as exact values whenever suitable.*
☒	☐	For Bayesian analysis, information on the choice of priors and Markov chain Monte Carlo settings
☒	☐	For hierarchical and complex designs, identification of the appropriate level for tests and full reporting of outcomes
☒	☐	Estimates of effect sizes (e.g. Cohen's *d*, Pearson's *r*), indicating how they were calculated

Our web collection on statistics for biologists contains articles on many of the points above.

Software and code

Policy information about availability of computer code

Data collection	Biotek Gen5 software was used for collection of the protein activity measurements. PR.ThermControl was used for nanoDSF data collection.
Data analysis	Microsoft Excel was used for the analysis of activity measurements. XSCALE and HKL2MAP were used to identify the Zn site and to phase the initial model. Iterative cycles of manual building were conducted in COOT50 with crystallographic refinement in REFMAC5. PyMOL 2.3 and UCSF Chimera 1.14rc softwares were used for structural alignment, analysis, secondary structure assignment and visualization of protein structures. PR.ThermControl was used for nanoDSF data analysis. Multiple sequence alignments were performed with M-Coffee from the T-Coffee software package and phylogenetic analysis was conducted with MEGA X. PROPKA was used to protonate the variants. TIP3P was used to solvate the structure with water. The Amber package of molecular simulation software and the ff14SB force field were used to perform the MD simulations. The Zn ions were treated with the Li-Merz parameters. The SHAKE algorithm was applied to bonds involving hydrogen atoms. The location of the binding pocket was tracked using 460 VMD. Hierarchical agglomerative clustering algorithm was performed in CPPTRAJ. Substrates were drawn and converted into a 3D structure with Maestro. Docking was performed with AutoDock as a docking engine and the DrugScore2018 distance-dependent pair-potentials as an objective function.

For manuscripts utilizing custom algorithms or software that are central to the research but not yet described in published literature, software must be made available to editors and reviewers. We strongly encourage code deposition in a community repository (e.g. GitHub). See the Nature Research guidelines for submitting code & software for further information.

Data

Policy information about availability of data

All manuscripts must include a data availability statement. This statement should provide the following information, where applicable:

- Accession codes, unique identifiers, or web links for publicly available datasets
- A list of figures that have associated raw data
- A description of any restrictions on data availability

All data generated or analysed during this study are included in this published article and its supplementary information files. In case "data available upon request" is indicated: the data were excluded from the manuscript due to space limitations. The data can be made available upon request. The structural data of Igni18 have been deposited at the PDB database under accession code 6HRG.

Field-specific reporting

Please select the one below that is the best fit for your research. If you are not sure, read the appropriate sections before making your selection.

- [x] Life sciences
- [ ] Behavioural & social sciences
- [ ] Ecological, evolutionary & environmental sciences

For a reference copy of the document with all sections, see nature.com/documents/nr-reporting-summary-flat.pdf

Life sciences study design

All studies must disclose on these points even when the disclosure is negative.

Sample size	For the PVRs definition analysis, only crystal structures with more than 220 aligned amino-acids or an RMSD value below 3 were taken. Only a representative structure was used for each characterized activity. For experiments concerning activity measurements, each measurement consisted of at least three replicates takes (n>3). Each experiment was repeated at least three times. As the standard deviations were low and internal controls consistent, this sample size was considered as sufficient.
Data exclusions	Data were not excluded from analysis.
Replication	All activity measurements were performed independently at least three times.
Randomization	Randomization does not apply to our study.
Blinding	Blinding does also not apply to our study.

Reporting for specific materials, systems and methods

We require information from authors about some types of materials, experimental systems and methods used in many studies. Here, indicate whether each material, system or method listed is relevant to your study. If you are not sure if a list item applies to your research, read the appropriate section before selecting a response.

Materials & experimental systems

n/a	Involved in the study	
x		Antibodies
x		Eukaryotic cell lines
x		Palaeontology and archaeology
x		Animals and other organisms
x		Human research participants
x		Clinical data
x		Dual use research of concern

Methods

n/a	Involved in the study	
x		ChIP-seq
x		Flow cytometry
x		MRI-based neuroimaging
